# Instructive roles and supportive relationships: client perspectives of their engagement with community health workers in a rural south African home visiting program

**DOI:** 10.1186/s12939-020-01377-z

**Published:** 2021-01-13

**Authors:** Christina A. Laurenzi, Sarah Skeen, Bronwynè J. Coetzee, Vuyolwethu Notholi, Sarah Gordon, Emma Chademana, Julia Bishop, Mark Tomlinson

**Affiliations:** 1 Institute for Life Course Health Research, Department of Global Health, Stellenbosch University, Tygerberg, South Africa; 2 Department of Psychology, Stellenbosch University, Stellenbosch, South Africa; 3 One to One Africa Children’s Fund, Cape Town, South Africa; 4 School of Nursing and Midwifery, Queens University Belfast, Belfast, UK

**Keywords:** Community health workers, Client perspectives, Client engagement, Relationship-building

## Abstract

**Background:**

Community health worker (CHW) programs have been positioned as a way to meet the needs of those who experience marginalization and inequitable access to health care, and current global health narratives also emphasize their adaptable nature to meet growing health burdens in low-income settings. However, as CHW programs adopt more technical roles, the value of CHWs in building relationships with clients tends to be overlooked. More importantly, these programs are often reframed and redeployed without attending to the interests and needs of program clients themselves. We set out to gather perspectives of program and CHW engagement from clients of a maternal and child health program in rural South Africa.

**Methods:**

We conducted 26 interviews with pregnant or recently-delivered clients of the Enable Mentor Mother program between February–March 2018. After obtaining informed consent, a trained research assistant conducted all interviews in the clients’ home language, isiXhosa. Interviews, translated and transcribed into English, were organized and coded using ATLAS.ti software and thematically analyzed.

**Results:**

We found that clients’ home-based interactions with Mentor Mothers were generally positive, and that these engagements were characterized by two core themes, instructive roles and supportive relationships.. Instructive roles facilitated the transfer of knowledge and uptake of new information for behavior change. Relationships were developed within the home visit setting, but also extended beyond routine visits, especially when clients required further instrumental support. Clients further discussed a sense of agency gained through these interactions, even in cases where they chose not to, or were unable to, heed their Mentor Mother’s advice.

**Conclusions:**

These findings highlight the important roles that CHWs can assume in providing both instructive and supportive care to clients; as deepening relationships may be key for encouraging behavior change, these findings pinpoint the need to bolster training and support for CHWs in similar programs. They also emphasize the importance of integrating more channels for client feedback into existing programs, to ensure that clients’ voices are heard and accounted for in shaping ongoing engagement within the communities in which these programs operate.

**Supplementary Information:**

The online version contains supplementary material available at 10.1186/s12939-020-01377-z.

## Introduction

Historically, community health worker (CHW) programs were conceptualized as a way to meet the needs of those who experience marginalization and inequitable access to health care. The 1978 Declaration of Alma-Ata, which formally laid the foundations for countries to initiate and mobilize CHW programs, was based on social justice, health equity, and empowerment of local communities and capacity [1]. However, as various low- and middle-income countries (LMICs) began to implement CHW programs, this equity-based approach to health service provision quickly became subject to debates around their financing, implementability, and societal value. In many countries, they were dissolved to make way for market-driven solutions to health care [2].

Currently, in the context of a global shortage of health care workers, and a rising demand for services, community-based approaches to health service delivery are being re-evaluated as a way to fill this gap [3]. Deploying CHWs today is viewed as a largely technical solution to poor health system coverage and quality of care as CHWs step in to assist health care providers fulfill essential tasks [4]. It is also a means for countries to achieve multiple ambitious targets as set out in the Sustainable Development Goals (SDGs)—such as SDG 3, which reads “Ensure healthy lives and promote well-being for all at all ages”—and is part of the current drive for Universal Health Coverage, which has been championed by the World Health Organization [5]. In many settings where health resources are limited, CHWs may be the quickest and most successful ways to reach these kinds of targets and meaningfully improve health outcomes at a population level.

However, while there is a renewed interest in ascertaining how to best engage CHWs to fulfill important roles in health promotion and disease prevention, the roles of CHWs are malleable and not easily-defined in a universal way. In most settings, CHWs are trained lay health workers—who operate either within or in conjunction with, or outside of the formal health (state) system, who are typically members of the communities in which they work, and who are seen as being able to reduce the burden on under-resourced and overstretched health professionals [6]. Part of reducing this burden comes through task-shifting: the systematic delegation of tasks from one cadre of health worker to another, often less professionalized, cadre [7]. In times of crisis, such as during the emergence of the HIV/AIDS epidemic in sub-Saharan Africa, task-shifting has been essential in expanding and re-purposing the workforce [8]. For CHWs operating across programs with different health focus areas, task-shifting has become the norm. As programs become more broad-based in their framing, there is also a risk of “task-piling” or “task-dumping” where CHWs take on increasingly more responsibilities previously completed by nurses or not at all [9].

This shift has ostensibly been driven by the needs of health systems, and not necessarily by the people served by CHW programs. There remains little consensus on what the beneficiaries, or clients, of CHW programs actually require and want from programs, as few studies incorporate their perspectives [10, 11], especially in LMICs [12]. As a result, there remains a stronger focus on evaluating delivery mechanisms and health outcomes than on the processes through which clients take up new information and connect with their CHWs [13]. While both areas of focus are vital, understanding client perspectives can be just as important as more commonly-measured health outcomes [14], in revealing priorities, capturing reasons for engagement or dropout [15], or contextualizing challenges in implementation.

The existing data we have from other clinical settings, and different types of health providers, indicates that relationships are important aspects of clinical encounters in multiple distinct ways. Qualitative research in particular has provided rich data on how clients experience, and respond to, health encounters [16, 17]. A systematic review on patient perspectives of quality of care across formal health delivery settings found that patients most commonly identified communication, access, and shared decision-making as important in clinical interactions [18]. This finding echoes work by health practitioners and researchers highlighting the importance of shared decision-making and improved communications as a means to facilitating a sense of respect and agency in patient-provider interactions [19, 20]. These relationships and experiences drive whether or not patients are motivated to continue to seek care, and ultimately have bearing on whether health outcomes can be improved, especially in LMICs [21].

Clients within CHW programs tend to be overlooked in existing research, and we do not know the extent to which they similarly benefit from, and rely on, relationships as a means to support them to achieve better health outcomes [22]. Given the community-embedded nature of the CHW role, however, we can hypothesize that these relationships are equally, if not more, important in these programmatic frameworks [23]. Without a more nuanced understanding of how clients experience and engage with their CHWs, we may be missing critical information about how to make these programs more effective, how to meet the needs of marginalized individuals, families, and communities, and how to ensure that shared decision-making is a reality in health programs.

### Addressing a critical gap

To build a more comprehensive picture of mechanisms through which CHW programs may bring about change, we need to ask clients themselves about their experiences with and perspectives on their CHWs, as the main interface with the program, and the relationships they maintain. This imperative is reflected in implementation science frameworks such as the Medical Research Council’s guidance on process evaluation for complex interventions [24]. Drawing on one facet of this framework, reflected under mechanisms of change—“participant responses to and interactions with the intervention”— our work aims to capture participant voices clearly and comprehensively. This paper aims to explore how clients in a maternal and child health program in rural South Africa experience their engagement with their CHWs.

## Methods

Ethical approval for this study was granted by Stellenbosch University’s Health Research Ethics Committee (N16/05/062). An additional file includes further ethical considerations and reflections from the authors.

### Design and setting

This study was descriptive in nature and utilized semi-structured qualitative interviews with pregnant women and recently delivered mothers who were clients of the Enable Mentor Mother program, further referred to as Enable. This study was linked to a larger study of the implementation of Enable during its first three years, part of ongoing efforts to strengthen the quality of care delivered to mothers and infants in the region where Enable operates [25, 26]. The O.R. Tambo District, where Enable is based, is among the poorest districts in South Africa. In addition to high unmet need for health services, many of Enable’s clients live in remote rural areas, where accessing health facilities can be challenging, and resources unevenly distributed [27]. Enable is the only program of its kind to operate in this specific area. With regards to maternal and child health, the O.R. Tambo District has significantly higher rates of maternal and neonatal mortality than the national average [27]. A large number of mothers and caregivers are able to access government child support grants, which many of them rely on for basic child care needs given high rates of unemployment and rural-urban migration [28].

### About Enable

Enable is a home visiting intervention focused on maternal and child health and nutrition; the program has been operating in the O.R. Tambo District since 2016. The program uses the Philani Mentor Mother model, which was originally designed and implemented by Philani Nutrition Project in peri-urban Cape Town [29]. In this model, Mentor Mothers (MMs) are identified by established community leaders as mothers who have managed to raise healthy children despite significant adversities. Recruited mothers undergo a six-week training, followed by an evaluation, before a subset are selected to work in their own communities. MMs recruit and follow-up on pregnant women, as well as specifically targeting families with underweight children, to provide supportive, preventive care in the home for up to five years. While Enable adheres to Philani’s model of intervention training and content, the program was adapted to be a “social franchise” of the original model, in which a model is applied in a new setting, supervised and implemented by a different organization [25]. While a MM may have up to 50 or 60 maternal clients, each client is enrolled and visited by only one MM, although these visits occasionally involve supervisors or program coordinators. MMs are distributed across a wide geographical area, each covering their own set of clients within a given village.

### Recruitment and sampling

A list of all clients in the Enable program was obtained from program staff. In order to ensure a diverse sample of interviewees, two clients per Mentor Mother were included as a target sample. The first author (CL) purposively sampled clients from each MM’s caseload, while ensuring variation in characteristics by age and number of children. Alternative clients were contacted in cases where a client was uninterested or unavailable.

### Data collection procedures, transcription and translation

Interviews were conducted between February and March 2018, and were arranged and completed by an isiXhosa-speaking researcher with extensive qualitative interview experience (VN). CL devised a draft interview schedule and met with VN to discuss each question and refine or clarify phrasing where needed. The semi-structured interview schedule was then finalized and used to guide interviews. Topics covered included: client experiences with their own individual Mentor Mother; knowledge gained since enrolling in the program; possible avenues for improving the program; and experiences with the larger health system. Informed, written consent was obtained before interviews began, and all interviews were conducted in participants’ homes and audio-recorded with consent. Both informed consent and the interview itself were conducted in participants’ first language, isiXhosa. Interviews averaged one hour duration each. Rigorous quality control measures were taken to ensure consistency and alignment with core questions. In debriefings following the interviews, CL and VN reviewed interviews, and were satisfied that the target number of participants allowed for saturation. Saturation was assessed by the presence of rich transcripts, with coherent information across interviews, and the understanding that additional information gathering would likely lead to redundancy in the data [30].

Between April and October 2018, an experienced isiXhosa-speaking team reviewed audio recordings, and simultaneously translated and transcribed interview content into English. Specific words or short phrases were left in isiXhosa on a case-by-case basis to preserve meaning, and alternative definitions or explanations were added where appropriate. CL met with the transcription team regularly to discuss progress. A senior member of the transcription team checked 50% of transcripts over the course of the transcription period for quality and accuracy.

### Data analysis

Thematic analysis was employed, using the methods described by Braun and Clarke [31, 32]. Data were organized and coded using ATLAS.ti qualitative software. All transcripts were read closely before coding for familiarization, and were later coded inductively [33]. The topics covered, while aligned with questions in the interview schedule, varied substantially among interviewees. A total of 98 codes were initially developed to summarize content across the transcripts. Following several rounds of collation, review and refinement, 51 codes formed the final code list. Initial themes were generated as described by Braun and Clarke to examine viable candidate themes, and these themes were reviewed against the dataset to ensure alignment, before final themes were defined, named, and narratively written up [32]. A second independent coder (SG) read three interviews (11.6% of interviews) and discrepancies were resolved through discussion between SG and CL.

## Results

Participants (*n*=26) were all female, and on average, 26.9 years of age (SD = 5.77) at the time of the interview. Nearly 60% of participants were enrolled as clients in the first six months of Enable’s implementation. Ten participants were first-time mothers (38.5%), and eight were married (30.8%).

The coded data were grouped into clear superordinate themes that clearly reflected clients’ perspectives on and extent of engagement with their MMs. Upon review, codes were collapsed into three primary theme groups (instructive, preventive, and supportive care). Many codes under preventive care, however, detailed participant responses to and reflections on the MM intervention. As such, these codes were distributed under two overarching themes. These themes included instructive roles (clients’ experiences with MMs in instructive contexts, and their reflections on this role) and supportive relationships (clients’ experiences with additional support, and reflections on MMs in these relationships). Examples are shared in Table 1. Client perspectives on the larger health system challenges have been reported elsewhere [34].

**Table 1:** Examples of codes and sub-themes under each overarching theme

Overarching theme	Sub-theme	Examples of codes that formed the sub-theme
Instructive roles: receiving new information for behavior change	Experiences of instructive encounters	Clear teaching style; breastfeeding information
	Reflecting on and engaging with instructional experiences: High-quality interactions fostering trust and credibility	Internalizing advice from MM; accountability to MM
	Reflecting on and engaging with instructional experiences: Facing challenges in enacting advice	Deciding not to follow advice; encouraging and problem-solving
Supportive relationships: building relationships and sharing burdens	Experiences of mutual respect and familiarity	Acceptance in client-MM relationship; approachable and relatable
	Reflecting on the scope of the MM role: Extending support beyond the job’s requirements	Commitment to client; connecting outside of typical visit
	Reflecting on the scope of the MM role: Limitations to the scope of the MM’s supportive role	MM challenges with confidentiality; limits to MM role

### Instructive roles: receiving new information for behavior change

In speaking about interactions with their MMs, most clients shared experiences about new information learned, internalized, and accepted, and how this information was relayed (i.e. the mode of instruction). Clients also reflected on the impact of this education—what it meant for their child’s health and their own sense of agency, as well as challenges to enacting this advice.

#### Experiences of instructive encounters

In their interviews, clients affirmed that learning new information about their own and their child’s health was a central part of the MM-client interaction. Clients emphasized the skills of their MMs in conveying new instructive information, and ensuring client understanding at regular intervals:

*PID13: When she arrives, talking to me, she explains it and asks if I heard what she said.*

*Interviewer: Okay, why it is important for her to ask you if you heard what she was saying?*

*PID13: It is important to me because I have to know it … Because it’s me who agreed to work with her, so I have to know it.*

In addition to sharing strategies for learning, clients indicated that they felt compelled to satisfy their obligations to the MM-client relationship.

Not all instruction was described as verbal. Clients highlighted the importance of hands-on activities and examples, saying they learned better when their MM acted something out:

*PID6: She also acts some other things, I mean she has got many examples … and her examples are clear and corresponding to what she is saying.*

When MMs supplemented their instructions and explanations with actions, clients described grasping new knowledge more quickly, and also were able to put it into practice. In certain cases, this also applied to deciding which information was most important. One client spoke about filtering information through cues from her MM:

*PID21: When she emphasizes something, she looks at you directly and says “hey, I’m serious about what I’m telling you, as you always like not to take things not seriously” so that is what I notice with her, and say hey, [my MM] emphasized this thing …*

These instructive strategies were often tailored to what a particular client needed to learn, and to ensure uptake of new information and thorough understanding.

#### Reflecting on and engaging with instructional experiences

Beyond sharing experiences and examples of receiving information from their MMs, clients also reflected on the quality of the interaction with their MM as they described the role this information played for them, and the context in which it was delivered. They spoke about perceiving their MMs as trustworthy and being accountable to them, about how they engaged with new health information, and about situations when they faced challenges to taking action in line with their MM’s advice.

##### High-quality interactions fostering trust and credibility

Speaking about the informational and instructive role of their MMs, clients compared their “current” selves to who, and how, they had been before being enrolled in the Enable program, detailing the process of gaining knowledge and confidence and the importance of having a trusted peer.

*PID1: As a person, you learn till you die, you see? So there are so many things that I learned. I didn’t know that you have to wash your hands before you touch a baby’s bottle, then wash the bottle and prepare food for the baby, I didn’t know that. Even feeding the child, I thought that you just feed the child … I didn’t know the motive behind it, you see … So after she came and explained to me about breastfeeding, telling me that breastfeeding is very important, even the scale [taking the child’s weight] is very important, telling me that the child must never miss the scale dates [at the clinic], I became alright, I saw myself as a good person and I did things the way she told me to.*

Another client spoke about adopting new information to make informed decisions, and why she considered her MM as a credible source of information:

*PID24: I mean like as a person what you are talking about becomes visible from your facial expression, you see from the face that, “okay, this person knows what she is talking about and she is living it” … She is living with what she is saying, you see … And [you] notice that hey, maybe this thing is good, so you rather try it and [it] helps you after trying it and you see that, no these things that are said by this person are good, so let me follow them and be close to her so that when she talks I do, every time she talks I do what she is saying.*

For some, this credibility also came with the MM’s approach, as one client described how her MM “*would give compliments saying ‘no my child you are listening carefully’ because she teaches those things in a good way. You will never understand someone who came to you with attitude*” (PID17). Clients also shared a renewed sense of external accountability, and anticipation, from having a monthly or bimonthly visitor who they respected:

*PID6: I once had that thing on my previous children … That thing of not going to the clinic. But having that mentality of there is no one who is going to ask me. Now I have that feeling, wishing that she can arrive quickly and also see that the weight of my child increased now, that time there was a scale.*

These reflections on assessing and internalizing information revealed clients’ abilities to engage with their MMs in an enabling space, which fostered important changes in their own knowledge and practice with their infant.

##### Facing challenges in enacting advice

These interactions also enabled clients to recognize their own agency, even when they did not fully follow the MM’s counsel. A young mother spoke about the strength she was able to draw from conversations with her MM:

*PID2: When I forgot, she reminds me and say “did you do what I told you” and if I didn’t do it I say no I didn’t do it “why you didn’t do it?” so deep inside that thing is encouraging me to continue doing it till I get used to it because it also helps me, at the end it’s me who will be in [trouble]. So by doing that, she is strengthens me because she is the one telling me things.*

Another client spoke about needing to make a decision about breastfeeding based on her own needs, despite understanding its benefits from her MM:

*PID25: What was challenging, it was when she asked me to breastfeed for six months but I couldn’t, because I was going back to school and I was going to leave him behind so I was not able to do that … yes, she told me it is good [important] but I couldn’t, and I was like, ‘she will forgive me on this one.’*

By acknowledging her MM’s ability to “forgive” her, this client also described a sense of respect in the relationship, indicating her MM would not condemn her for her decision. Other clients similarly shared challenges to enacting certain advice from their MM due to financial or logistical constraints. Responding to a question about barriers, one client spoke about lacking funds for medicine recommended by her MM:

*PID22: I don’t fulfil some of the things she teaches me … it is on things that we need to eat [medicines we need to take] when we are not well, so I am unable to reach them because there is no money.*

Clients facing these situations might try to enact more feasible options, but continue to face constraints related to poverty.

### Supportive roles: building relationships and sharing burdens

In describing and reflecting on their relationships with their MMs, clients also revealed how the Enable program provided a deeper sense of social support, through the mutual respect, responsiveness, and attentiveness of their MMs. This relationship, built over time, was described as valuable and distinct from other types of relationships.

#### Experiences of mutual respect and familiarity

Sharing their experiences with MMs, clients iterated that respectful, supportive interactions were essential to their perspective of the program and their MM relationships. They described supportive qualities that their MMs practiced in typical engagement. Consistency and continuity were highly valued:

*PID24: She is not a person that changes having different faces every day, she remains the same, and she is always humble and I can ask anything that I want to ask from her. She is just a person like that, I can even call her and ask how is this going, what must I do now.*

In addition to experiencing support through warm interactions with MMs, clients spoke of knowing a MM through her community embeddedness—reiterating a sense of security, trust, and familiarity that comes with health workers operating in their own communities.

*PID5: Since we know her, she is from here, when we have something that we do not understand, we just talk like [MM name], what it is about a certain thing, just like that because we know her. If you can come as another person [from outside the community] maybe we can have things that we are afraid of, but we are not afraid of her.*

Where this support was tangible, it further affirmed the role of a MM, showing how she was able to build on existing trust with additional resources.

*PID17: She is diligent to this job but even before she started doing this job she was diligent, she cannot manage to watch someone suffering, even if it’s not her family member, that is her nature … most families here are getting social grants because of her.*

The supportive qualities revered by clients centered on more personal, community-based attributes, facilitating their acceptance of the MM program.

#### Reflecting on the scope of the MM role

Clients also reflected on the importance of having a supportive peer and health paraprofessional in their lives. Many women described a tangible feeling of support that extended beyond what they saw as the purview of the MM; a few women were stricter about drawing boundaries around the role of the MM. These reflections centered on the individual MM’s actions, and did not cover higher-level support from Enable’s supervisors which was also part of the program.

##### Extending support beyond the job’s requirements

One client described a surprise visit from her MM, who arrived on her day off to check on the client’s progress following a health complication:

*PID11: Hey she once surprised me, she came on a Saturday the time my stitches were damaged, I called her Friday evening and she came, shame, on her off-duty day, she was telling me that “I’m not working but I will come just for your sake”.*

*Interviewer: How did you feel about that, like it was not her day on duty but still when you are in trouble she comes to help you?*

*PID11: It’s kind of...I took her as someone who is dedicated to her work, she does not have that “I’m supposed to be doing my things” and all that, but she can sacrifice her time and do what she [is] supposed to be doing.*

Another client echoed a similar sense of feeling supported outside of typical visits, with a broader household audience absorbing her MM’s advice:

*Our Mentor Mother is very diligent, I love her … it doesn’t matter where she is, if she is at work, she would say after work I will come and listen to what you are calling me for, here at home we like inviting her even if we just sitting we will just call her (PID14).*

These interactions showed a willingness on the part of the MM to work outside the structure of her job, and to put time into fostering positive relationships, even if it meant sacrificing her own time—something that clients felt humbled by.

For particularly vulnerable clients, extended support could act as a lifeline. One young mother who lacked a broader family support system stated, “*I can say she is more like a parent to my child more than me*.” When prompted further, she explained:

“*Because she can advise and I know nothing about a child, saying this is how to take care of a child, telling me about the stages of a child, telling what to feed a child when a child is at which stage and breastfeeding when she was still breastfeeding*” (PID2).

Another young mother, who was pursuing her further education away from home without any support from her child’s father, described the emotional support her MM provided:

“*That is what I need, I need somebody, when I cry I feel like there can be someone to give me something positive, as I’m negative and she is always positive*” (PID11).

Despite the high level of poverty in the program catchment area, some clients were worse off than their peers because of lack of support, child disability, or living in extremely remote households. One client, who cared for a disabled child completely on her own, shared the extent of her MM’s support:

*PID26: She would say, ‘never lock yourself with poverty alone here, when you have nothing [to cook] you must phone.’*

*Interviewer: Okay, how does that make you feel, someone saying such things to you?*

*PID26: I feel happy sister, because these days there is no one who can care for you like that.*

This client and a number of others reflected on times when their MMs provided important assistance when clients were in desperate circumstances—reiterating that this support sometimes took on an instrumental role beyond the emotional and informational roles that MMs played.

##### Limitations to the scope of the MM’s supportive role

In a few cases, clients were less inclined to seek additional support from their MMs. These individuals were aware of limitations of MMs’ roles, or seemed to question the extent of MM confidentiality while also being embedded in the same community. One client in particular spoke about the differentiation between official and unofficial “duties”, noting that even if she were to mention specific challenges in passing to her MM, this would not constitute part of the visit. When asked about specific stresses she might discuss, she noted:

*PID1: She took care for us while we were pregnant and she is still taking care of us as we have children, so I just think those stress issues are just … it’s just a talk after she finished her duties, you see.*

*Interviewer: Okay.*

*PID1: Like telling her that me and my husband we had a problem and I was hurt here and there, so just a talk … It’s not part of her work when I think.*

Even if these conversations took place, this client did not feel her MM was any more able to counsel her on these kinds of challenges than a friend might be. Some clients also felt that there was a limit to the support they could seek from MMs, even if confidentiality was discussed, as they came from the same small, tight-knit community. One client described a hypothetical situation:

*PID24: So I would share my problem with you, but since you know me you share it to your loved one, and that one share to another one … So that is what tells me that my problem is my problem, I cannot share it to a Mentor Mother.*

These perspectives about how MM roles might be limited reflected a sense of caution and care from clients, and highlighted diverse approaches to the client-MM interaction.

## Discussion

We explored clients’ experiences of a home visiting program for maternal and child health to gather perspectives on the program, and more specifically, on clients’ relationships with their Mentor Mothers. This analysis helps to identify potential areas for strengthening programming from the perspective of those receiving services directly. It also offers a chance to consider the trade-off between accomplishing tasks and cultivating relationships in similar CHW programs.

### Client engagement as a window to behavior change

Overall, client perspectives were positive and reinforced the aims of the Enable program. Clients shared reflections on having an ongoing relationship with their MM, and what this engagement meant for their health and wellbeing. These relationships were central to their experience of Enable, and clients described ways in which these relationships motivated them through a combination of education and encouragement—helping increase their confidence in health-related decision-making. These findings on supportive relationships and enabling encounters map onto existing understandings of how lay health worker interventions activate behavior change [35–37], as well as how shared decision-making and client-centered communication in clinical encounters can engage clients as agents in their own health [20, 38, 39]. They also speak clearly to the World Health Organization’s framework of integrated people-centered health services [40] which echoes the need for “care delivered in an equal and reciprocal relationship” (p. 4).

These perspectives also raise the question of how, and why, these kinds of relationships and interactions are possible in community health settings. Prior research on CHWs has identified obvious advantages that community members may have in enacting these roles: these relationships can take place outside of clinical hierarchies, with someone from a similar or same community [2]. They may also provide more individualized, thorough care in non-formal health settings [41]. As these data indicate, the supportive nature of the relationships between home visitors and clients may be able to create a mechanism for bidirectional accountability, by fostering a sense of mutual respect and responsiveness, and encouraging clients to work towards agreed-upon goals [42]. These relationship qualities are far removed from more stressful, power-imbalanced interactions in formal health facilities that have been reported in many rural and LMIC settings [43].

At the same time, these relationships can also be fraught, as CHWs are tasked with navigating tensions between formal and informal modes of work, and similarly, with projecting a certain image as a professional while also maintaining a degree of relatability [26]. It is evident that many clients appreciated their MMs’ flexibility and willingness to meet diverse needs in their “supportive” roles. However, it is also important to think about how implementers make decisions about how to most appropriately invest in their relationships with clients, so that both types of individuals’ needs are being met.

Importantly, not all relationships were described as equally positive. A small subset of clients shared reservations about their MM, related to confidentiality concerns or lack of clarity around the scope of the program. It is also important to note that not all CHWs are equally committed, or receptive to training, and that some CHWs may struggle to build durable, reciprocal relationships with all of their clients [44]. Furthermore, even when the scope of a MM’s role is well-defined, clients themselves may elect to engage in a more bounded, professional relationship with home visitors.

These client responses can also be seen as a strength of the program model—enabling clients to exercise agency, challenge advice that does not work for them, and find ways to compromise where possible. This was evident even in data from clients who spoke warmly about their MMs, but who faced additional barriers to adopting different strategies. For some mothers, fully taking on their MM’s advice was more challenging in their specific circumstances, and not always an act of resistance or reluctance. That clients sometimes did not follow their MM’s advice could be seen as contrary to the program’s aims, by stalling progress towards certain health goals. In some cases, this was out of necessity, not choice. However, these modes of engagement are also critical for empowering clients to find their own solutions, especially when evidence-based program goals confront context-related constraints.

### Listening to clients

This analysis adds an important perspective, largely missing from the literature on CHW programs and task-shifting, by incorporating the voices and priorities of clients. Clients’ perspectives provide insight into the kinds of services and interventions they prioritize, and thus offer a way to map these priorities against programmatic goals. For instance, supportive relationships are often seen as a means to achieve intended program outcomes in lay health worker interventions [12]. However, we found that they also provide their own form of intervention through linking clients to broader support networks beyond the health-specific aims of the program. The supportive relationships that clients described were, often, more deeply personal stories about how MMs provided information that made them feel capable of raising an infant alone, or able to rely on another person when they were in desperate circumstances.

These findings also suggest that even when information and activities are present and prioritized by intervention designers, supportive relationships form an equally important part of these programs, and the ability to draw on existing bonds can make programs more effective. Clients’ characterizations of these relationships, and how they engaged their MMs over time, provides fresh insights into how evidence-based community health programming can and should draw on principles of equity and respect in theorizing how behavior change can happen. Interestingly, clients did not often draw on experiences where Enable’s supervisors intervened to connect them to more tangible resources through additional referrals or connection to government-provided social services. Instead, interviews tended towards a narrower focus on the nature of the MM-client relationship.

These findings fill some gaps in concurrent research published by our team, with the Enable program, on MM-client relationships [25]. Our team obtained consent from clients and MMs to audio-record individual home visits with 85 clients, and analyzed recordings using a novel communication skills checklist capturing existing communication skills. While skills showcasing active listening and interactive delivery skills were frequently observed in visits by all MMs—skills that, in this analysis, would be more closely linked with fulfilling instructive roles—communication skills related to “connecting” and relationship-building were less common. The qualitative client interviews in the present analysis, however, suggest that the MM’s supportive role is highly valued by clients, if not universally observable across a cross-section of home visits. Many of the interviews pointed to non-verbal cues and other “connecting” factors contributing to relationship building that are not as easily captured in audio recordings. As such, triangulating diverse sources of data and/or adopting a mixed methods approach for future studies may provide more contextualization and a closer read of these experiences at the household level.

Relatedly, this analysis raises interesting questions about fidelity and adherence to the program: as noted above, clients did not always listen to their MMs, nor did MMs always adhere strictly to their job description. While measures of fidelity and adherence are sometimes used to explore the effectiveness of CHW programs, qualitative data may be able to highlight where these defined measures can fall short. Clients were clear that their relationships were able to provide them a sense of agency and collaboration in the routine visits. Less evident, but still present in these findings and in other related interviews, was the flexibility that MMs themselves employed to support clients and build trust—even when these actions occurred outside of the bounds of their job’s expectations [26]. Other research has documented this flexibility and adaptation that CHWs use to support their client relationships [45].

### Implications for practice

These data, directly gathered from clients of the Enable program, offer insights into how to tailor specific delivery aspects of this particular program. They also provide lessons for wider-ranging programming and policies surrounding adapted models of community health programs in LMICs, specifically attuned to implementer-client relationships, while acknowledging existing tensions.

Firstly, our findings point to implications for training and program delivery. Programs that leverage a more complex range of skills, including counselling skills, in training have been found to produce better quality of care [46]. Working with populations in low-resource settings may also require a broader set of support-related skills and more flexible, responsive solutions in the field; these skills can be established by training focused on improving CHWs’ self-efficacy in developing relationships [47], and sustained through experience and supportive supervision [48]. While a review of CHW training found that curricula frequently covered interpersonal skills and managerial skills (including relationship building), training processes were found to vary widely, in contrast to high standards set for other health care professionals [49]. Especially in LMICs, where resources may be less available and CHWs may be shouldering additional expectations, training should focus on building foundational skills and responding to specific contextual challenges. This may entail a shift away from highly-technical content in the interest of developing more widely applicable interpersonal and logistical skills. Prioritizing high-quality, routinely practiced skills related to implementer competence in training settings is imperative as CHW programs continue to grow [50].

Secondly, these findings indicate the need to integrate more regular client feedback through channels that can feed into practice. While MMs typically provide individualized services to different clients based on need and ability, it may also be important for MMs to communicate the scope of their possible role and set boundaries from the time of client enrollment in the program [51]. This practice may prevent later confusion or misaligned expectations. For those clients who attempt to implement changes but are financially unable to do so, additional support may be needed to pick up these cases and help clients identify solutions. Even if MMs are aware of client challenges on an individual basis, common challenges might be able to be identified through support groups, community forums, or health events. From a research perspective, community-based participatory research may present a more comprehensive way to address disparities and empower program beneficiaries in implementation research, while simultaneously producing more effective program content [52]. These methods present further possibilities for broader kinds of stakeholder engagement as programs are newly developed and expanded. Collaborative processes, designed to ensure that a program meets beneficiaries’ and implementers’ needs, are emerging as critical in intervention development, adaptation, and evaluation [53, 54]. These processes include concepts of human-centered design and community advisory panels; they may be especially important for ensuring social franchises, for example, can be directed by users’ opinions and needs. However, more research incorporating these methodologies and specifically engaging clients in their own health is needed.

## Conclusions

Our study found that clients of a maternal and child health home visiting program formed relationships with their home visitors based on instructive, supportive care. We also found that most clients also felt a sense of agency and confidence from these interactions, even when they were unable or unwilling to fully adhere to health counsel. As community-based health programs are increasingly relied on for increasing access to health care, conscious decisions to solicit the perspectives of clients should be prioritized to maximize the potential of these programs.

## Supplementary Information

**Additional file 1.**

## Data Availability

The data used and/or analyzed during the current study are available from the corresponding author on reasonable request.
