# Supplementary material for: Instructive roles and supportive relationships: client perspectives of their engagement with community health workers in a rural south African home visiting program
Source: Int J Equity Health. 2021 Jan 13;20:32. doi: 10.1186/s12939-020-01377-z (PMC7805205; doi:10.1186/s12939-020-01377-z)
Supplement: Supplementary file 1 — Additional file 1. [file 12939_2020_1377_MOESM1_ESM.docx]

### Supplemental file: Note on researcher background and reflexivity

### Note: This is adapted from a chapter of the lead author’s doctoral dissertation and as such excerpts of this file may be found elsewhere online.

### Considerations for ethical community-based research

This study’s methodology was informed by the following overarching principles for ethical community-based research: transparency, non-interference, and knowledge dissemination.

All interviews were conducted in participants’ home language, isiXhosa, to ensure that participants were able to engage fully with the questions, and share responses, in the language in which they were comfortable communicating. Furthermore, as explained in the manuscript, an informed consent process (again conducted in isiXhosa), was followed each time individual participant data was collected.

In planning and arranging data collection, the lead author (CL) strove to pose minimal interference with the Enable Programme’s usual operations. Interviews were scheduled through a transparent channel of communication with Enable Programme supervisors. The research assistant (VN) visited each client ahead of her scheduled interview to discuss the interview process informally, and prepare her about what to expect regarding the informed consent and interview itself on a subsequent date.

At the end of data collection and analysis, CL returned to Nyandeni and held a dissemination day meeting with all MMs and staff in July 2019, which included a number of new staff members who had joined Enable subsequent to my research. CL presented a general set of de-identified findings using a slideshow and portable projector, giving opportunities to ask questions and discuss themes. Following the presentation, the supervisors and CL set aside time to engage the staff in discussions about ongoing challenges and needs. Salient findings were further shared with Enable’s senior management through routine reports and the final Phase 1 project report submitted in mid-2019. In the study team’s proposal to work with Enable for the second phase of their funding, we were able to integrate key findings and challenges raised during the research into ideas for collaborating in this next phase—thus strengthening the quality of Enable’s implementation as it continued to expand into new neighbouring areas.

## *Reflections from the lead author (CL)*

In this supplemental file, I include some reflections to contextualise my role in the research and data collection in particular. My first visit to South Africa was as an intern for the Philani Nutrition Centres Trust in Khayelitsha from June - August 2012, as an undergraduate studying Global Health and Health Policy as well as Politics at Princeton University. The following year, in July 2013, I moved to Zithulele, in the rural Eastern Cape, to begin a one-year fellowship working with Philani and Zithulele Hospital. In co-managing a cohort study following infants for the first year of life, I planned daily logistics, oversaw a team of female Xhosa data collectors, and conducted data quality control. This experience gave me both foundational research skills but also a closer understanding of Xhosa culture and the realities of living in a remote, rural area. I learned much about the challenges that mothers and their infants faced in accessing health care, absorbing the details of their lives through many hours of poring over questionnaire responses, and I also spent ample time accompanying and conversing with data collectors on field visits in participants’ homes and occasionally at government-funded public health clinics.

This prior experience—both with the Philani home visiting model, and in a rural area that resembles Nyandeni and is 19 kilometres from it as the crow flies—was formative. It was also essential to the insight that I was able to bring to this research process, having come from a privileged and very different upbringing. Over the course of my doctorate, of which this manuscript forms a part, I made 11 visits over two and a half years, ranging from five to ten days each, to spend time at Mankosi and in the surrounding areas with the programme. These visits were in my capacity as a project manager as well as for my research, and I attended monthly meetings, co-led trainings, and reviewed case files over these visits in addition to managing the research. Throughout this engagement with the programme, I gained a better understanding of some of the dynamics of implementing community-based programmes in this area.

My co-author for this manuscript, VN, conducted all interviews with the informants in this thesis, and I decided not to accompany her during this process. The language barrier was one reason for this decision, and as a non-Xhosa person, I did not want to feel as if I was present without being able to contribute or engage on a deeper level. While senior managers and even funders do accompany MMs from time to time, I did not want any information to be compromised, and did not feel it was useful or necessary for me to accompany VN from a supervisory standpoint. VN, with approximately a decade of experience in research, had been born and raised in a different part of the Eastern Cape, but was able to easily bridge worlds between researcher and participant. As such, I was confident in her ability to thoughtfully and meaningfully engage with participants, and we had regular discussions about how to approach certain questions, translate certain concepts, and manage unforeseen challenges in participant rapport.

While these considerations were more pressing for home visit audio recordings and client interviews, I could have more easily sat in on interviews with MMs. However, I again felt that having some distance could allow MMs to open up more easily. Having worked closely with this first cohort of MMs over the course of the project, I wanted them to feel comfortable speaking with VN without any pressure and in their own language. While the MMs were all familiar with VN, she was very much separate from their management and oversight, whereas I reviewed case files as part of my supporting monitoring & evaluation role with project.

These decisions were also made with explicit understanding of my role as a white, highly-educated American woman. I wanted to be aware, from the start of this work, of how my background and identity might affect the way I approached and framed my research. I also wanted to ensure that I engaged in respectful and mindful research practices along all phases of this doctorate. I first realised in Zithulele that the research participants with whom I interacted did not readily distinguish between me and other white South Africans, and I was reminded of this fact again in my interactions with Enable’s MMs. While this may have granted me a sense of being seen as more of an ‘insider’ than being seen as a foreigner, in many ways, this lack of distinction served as a reminder of the vast inequalities by race, culture, language, and socioeconomic status that are present in South Africa today.

The prior experiences I describe above enabled me to become more familiar with aspects of South African culture specific to the Eastern Cape and area in which I was working. I feel that this extended time in the area lent me a more nuanced perspective that I was able to bring to this work, which was an advantage. However, it is clear that my upbringing in a mostly-white affluent suburb in New Jersey limited the depth of my connection with and understanding of this context. The inherited systemic and intergenerational disenfranchisement of black South Africans has been perpetuated through inequitable research partnerships and programming, among many other ways. I acknowledged this power dynamic and also sought to mitigate further harm throughout this process.

As a researcher entering the field with these privileges, I wanted to be sure that my presence in these interviews would not be assumed; that my research questions or intent would not be exploitative or seen as such; and that my perceptions or analyses of the data could be done both systematically but also with a sense of cultural understanding. I read research outputs from similar community-based health research consistently throughout the period of my doctorate, and regularly reflected on my time in Mankosi through personal writing and many conversations. My continued communication with VN, as well as the members of the transcription and translation team, were essential in this regard. By working closely with VN, she and I were able to ensure that this research was mindfully conducted, and I am confident that participants did not feel unduly pressurised to participate. Similarly, I devoted time to building and maintaining a positive working relationship with VN and the rest of the team, but also sought to acknowledge and lessen the power dynamics and balance of responsibilities between and among us to the best of my ability.
